# Seroprevalence of SARS-CoV-2 specific Immunoglobin G antibodies in rural population of Western Maharashtra, India

**DOI:** 10.7189/jogh.13.06011

**Published:** 2023-04-21

**Authors:** Dhiraj Agarwal, Rutuja Patil, Sudipto Roy, Harshpreet Kaur, Sanjay Mehandale, Ashish Bavdekar, Harish Nair, Sanjay Juvekar, Girish Dayma, RESPIRE Collaboration

**Affiliations:** 1 Vadu Rural Health Program, KEM Hospital Research Centre, Pune, India; 2 Indian Council of Medical Research, New Delhi, India; 3 Hinduja Hospital, Mumbai, India; 4 Paediatrics Department, KEM Hospital, Pune, India; 5 Centre for Global Health, Usher Institute, Edinburgh Medical School, University of Edinburgh, Edinburgh, Scotland, UK; 6 Community Health Research Unit, KEM Hospital Research Centre, Pune, India

## Abstract

**Background:**

Severe acute respiratory syndrome coronavirus 2 (SARS-CoV-2) infection, responsible for the coronavirus disease 2019 (COVID-19) pandemic, has been a major public health concern requiring continuous efforts for understanding its epidemiology. Patients infected with SARS-CoV-2 have a wide range of clinical features ranging from asymptomatic infection to mild or severe illness with fatal outcomes or recovery. Population-based seroepidemiological studies are an effective method for measuring the rapid spread of SARS-CoV-2 and monitoring the pandemic’s progress.

**Methods:**

We conducted repeated cross-sectional community-based sentinel surveillance between January and June 2021 in the rural parts of the Pune district of Maharashtra, India to assess the seroprevalence against SARS-CoV-2 in three age categories. We selected 30 clusters for each round using a proportional population sampling method and 30 individuals in each of the three age groups (1-17 years, 18-49 years, and ≥50 years). We took blood samples from consenting study participants to check for the presence of Immunoglobulin G (IgG) antibodies against SARS-CoV-2 in all five rounds.

**Results:**

We included 14 274 individuals across five rounds; 29% were from the 1-17, 39% from the 18-49, and 32% from the ≥50-year-old group. Overall seroprevalence combining all rounds was 45%. There was an increase in seropositivity in rounds four (51.15%) and five (58.32%) contributed mostly by adults. We found that about 72% of elderly individuals ≥50 years in round five were seropositive. The factors strongly associated with the seropositivity were being in contact with suspected or confirmed cases of COVID-19 (odds ratio (OR) = 7.15; 95% confidence interval (CI) = 4.2-12.14), receiving at least one dose of COVID-19 vaccine (OR = 3.13 (95% CI = 0.70-14.07), being aged ≥50 years (OR = 1.97; 95% CI = 1.81-2.15), and being in an occupation belonging to a high-risk category (OR = 1.92; 95% CI = 1.65-2.26). Among 135 hospitalizations reported due to COVID-19-like illness, 91 (67%) were in the elderly age group of ≥50 and 33 (24%) were in the 18-49-year-old age group.

**Conclusions:**

Seroprevalence of SARS-CoV-2 was high in the last two rounds (April to June 2021) which coincide with the second wave of the pandemic (Delta variant B.1.617.2) in India. Overall, one in three children and one in two adults had antibodies for SARS-CoV-2. The suspected or confirmed case of COVID-19 emerged as the significant factor strongly associated with the seropositivity followed by COVID-19 vaccination.

The rapid global spread of the coronavirus disease 2019 (COVID-19) challenged the public health surveillance and response systems of most countries in detecting, tracking, and containing the transmission of severe acute respiratory syndrome coronavirus 2 (SARS-CoV-2) infections [1], which have led to 6.5 million deaths, with over 610 million confirmed cases by August 2022 [2]. India, the second most populated country in the world, with a high population density that presented an increased risk of COVID-19 infection, reported its confirmed COVID-19 case on 30 January 2020 [3]. Existing socio-economic disparities and high population density in the country have caused a high burden of confirmed COVID-19 cases [4].

Seroprevalence estimation is an important tool for monitoring SARS-CoV-2 transmission [5,6]. Reports show that 5%-80% of the infected patients may be asymptomatic and will not be detected during clinical diagnosis, and that they continue to spread the infection in the community and are responsible for the substantial spread of the disease. Studies suggest that, in some cases, people do not develop symptoms even after getting infected with SARS-CoV-2; simultaneously, such asymptomatic individuals exhibit a potential for viral transmission and a viral load equal to that of symptomatic individuals [7,8]. The seroprevalence studies conducted in Asia show that 80%-90% of seropositive individuals did not report any COVID-19 symptoms [9,10]. Thus, seroprevalence estimation plays a vital role in understanding the true extent of the spread of SARS-CoV2 infection which is necessary to build an effective public health response to COVID-19 [11,12].

As hospital-based surveillance is likely to miss asymptomatic and mild cases, the WHO global research map for COVID-19 recommends conducting population-level seroepidemiological studies to generate data on levels of infection in populations and adjusting containment measures accordingly [13-16]. As the pandemic progressed, large cross-sectional serosurveys have been conducted worldwide, including in India, which demonstrated the changing burden of SARS-CoV2 infection [17].

However, most serosurveys have been conducted in the adult populations residing in urban areas in different geographical regions within India with limited large-scale systematic evidence for rural populations and the paediatric age group.

We conducted repeated cross-sectional serosurvey in the rural population of Western Maharashtra in individuals above one year of age to estimate the extent of the spread of SARS-CoV-2 infection in the community.

## METHODS

### Study area and population

We conducted the study in two rural administrative blocks of the Pune district (Ambegaon and Junnar), which are 75-80 km away from Pune city and have approximately 6 25 700 inhabitants. The study area encompasses semi-urban, rural, and tribal populations, with agriculture and industrial labour being the most common occupations.

### Sampling strategy and study participant recruitment

We conducted repeated cross-sectional surveys for five through a cluster sampling method in the same geographical area (but not necessarily the same individuals each time). We considered a village to be a cluster and about selected about 30 for each round using a proportional population sampling. We then recruited 90 individuals (30 individuals in age group (1-17 years, 18-49 years, and ≥50 years) from each cluster. We collected the data for all five rounds from January 2021 to June 2021. The study team selected the centre of the village (cluster) as a starting point for data collection and visited the adjacent household to enrol the consenting participants.

### Sample size

With an estimated 1% seropositivity, absolute precision of ±0.5%, confidence level of 90%, and design effect of 2, we calculated the sample size for each round of survey to be 2160, and assuming 25% non-response rate with 30 clusters × three age groups ×30 individuals per age group, we included 2700 individuals per round.

### Clinical data collection

We collected information on basic demographic details and medical history (including clinical history with details of comorbidities, exposure history to laboratory-confirmed COVID-19 cases, symptoms suggestive of COVID-19 in the preceding month), as well as information about travel to known “hotspots” for SARS-CoV-2 infection and COVID-19 vaccination history. The data were collected using an Android-based application developed in-house on the Survey solutions® platform; the application had in-built quality checks which flagged errors in data. To further ensure data quality, field-level supervisors visited 10% of the households daily and confirmed the pre-collected information.

### Collection of blood samples and processing

We collected a blood sample of 2 mL from each participant under safety precautions and transported it to the site laboratory at 2-8°C for further processing. We conducted the serum separation within 24 hours at 3000 rpm (RPM) for 10 minutes at 4°C. Two aliquots were prepared, labelled, and stored in a deep freezer at -80°C until further processing.

### COVID-19 IgG testing

After the completion of each round, the stored sera were transported to the XYCTON Laboratory, Bangalore on dry ice and were tested for IgG antibodies against SARS-CoV-2 by using SARS-CoV-2 CheX. SARS-CoV-2 CheX is a qualitative ELISA for the detection of IgG antibodies to the Receptor Binding Domain (RBD) of Spike protein of SARS-CoV-2 in human serum.

### Community engagement

Before and after the initiation of the study, community engagement meetings were held in each study cluster (n = 150) with community representatives, including the Sarpanch (head of village), Gram Sewak (administrative head), accredited social health activist (ASHAs), Gram Panchayat members, and auxiliary nurse midwives (ANMs). To avoid any stigma or panic, we explained to the participants at the time of consent the serological test planned in this study, that it is not a diagnostic test, and that the test result do not differentiate between recent or past infections. The field research assistants (FRAs) and the study doctors distributed the reports and counselled the participants about the interpretation of the results.

### Data analysis

We extracted the anonymized clean data set for analysis in the STATA 15 (StataCorp, College Station, TX, USA). The primary outcome was to estimate the overall and age-specific (1-17 years, 18-49 years, and ≥50 years) seroprevalence of SARS-CoV-2 antibodies in the general population. We expressed the seroprevalence of SARS-CoV-2 as percentage prevalence for each age strata and overall, across all the five rounds of surveys. The secondary outcome was to assess the risk factors for SARS-CoV-2 infection including gender, occupation, and age, for which we used univariate logistic regression analyses.

### Ethical consideration

The KEM Hospital Research Centre institutional ethics committee (Ref No. KEMHRC/RVM/EC/524), the Academic and Clinical Central Office for Research and Development (ACCORD), and the Edinburgh Medical School Research Ethics Committee (Ref No. 20-EMREC-014) reviewed and approved this study. It was implemented following the National Ethical Guidelines for Biomedical and Health Research involving Human Participants issued by the Indian Council of Medical Research in 2017 and the recent National Guidelines for Ethics Committees Reviewing Biomedical & Health Research During COVID-19 Pandemic issued in April 2020 [18,19]. All participants who signed the consent or assent form (as applicable according to age group) were included in the study.

## RESULTS

### Demographic characteristics

**Figure 1:**
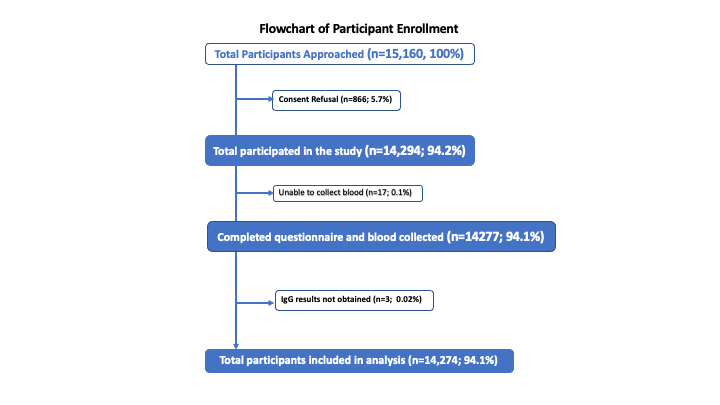
Flowchart of participant enrolment.

We approached 15 160 individuals, of whom 14 294 consented to participate in the study. Seventeen individuals refused participation at the time of blood collection, while IgG testing could not be performed for three participants due to haemolysis. Finally, we included 14 274 individuals in the analysis (**Figure 1**). The study population included almost equal gender representation, with 52% males. Regarding the age distribution, 29% of the participants belonged to the 1-17, 39% to the 18-49, and 32% to the ≥50-year-old age group (**Table 1**).

**Table 1:** Population characteristic

Characteristic	Round 1 (24 January 2021 to 20 February 2021), n (%)	Round 2 (21 February 2021 to 20 March 2021), n (%)	Round 3 (21 March 2021 to 24 April 2021), n (%)	Round 4 (25 April 2021 to 5 June 2021), n (%)	Round 5 (6 June 2021 to 26 June 2021), n (%)	All rounds, n (%)
**Age in y**	2857 (100)	2825 (100)	2854 (100)	2866 (100)	2872 (100)	14 274 (100)
1-17	844 (29.54)	898 (31.79)	826 (28.94)	769 (26.83)	794 (27.65)	4131 (28.94)
18-49	1075 (37.63)	1016 (35.96)	1122 (39.31)	1200 (41.87)	1192 (41.50)	5605 (39.27)
≥50	938 (32.83)	911 (32.25)	906 (31.74)	897 (31.30)	886 (30.85)	4538 (31.79)
**Gender**	2857 (100)	2825 (100)	2854 (100)	2866 (100)	2872 (100)	14 274 (100)
Male	1459 (51.07)	1517 (53.70)	1539 (53.92)	1432 (49.97)	1455 (50.66)	7402 (51.86)
Female	1398 (48.93)	1308 (46.30)	1315 (46.08)	1434 (50.03)	1417 (49.34)	6872 (48.14)
**Education**	2797 (100)	2788 (100)	2743 (100)	2706 (100)	2717 (100)	13 751 (100)
No schooling or less than primary	728 (26.03)	775 (27.80)	606 (22.09)	495 (18.29)	607 (22.34)	3211 (23.35)
Primary	673 (24.06)	527 (18.90)	482 (17.57)	570 (21.06)	610 (22.45)	2862 (20.81)
Secondary (middle and higher)	1227 (43.87)	1375 (49.320	1455 (53.04)	1501 (55.47)	1393 (51.27)	6951 (50.55)
Graduate	131 (4.68)	93 (3.34)	153 (5.58)	109 (4.03)	92 (3.39)	578 (4.20)
Postgraduate and professional	38 (1.36)	18 (0.65)	47 (1.71)	31 (1.15)	15 (0.55)	149 (1.08)
**Occupation.**	2797 (100)	2788 (100)	2743 (100)	2706 (100)	2717 (100)	13 751 (100)
Occupation with lower risk of exposure to COVID-19*	874 (31.25)	948 (34.00)	844 (30.77)	795 (29.38)	781 (28.74)	4242 (30.85)
Occupation with medium risk of exposure to COVID-19†	1758 (62.85)	1663 (59.65)	1726 (62.92)	1788 (66.08)	1820 (66.99)	8755 (63.67)
Occupation with high risk of exposure to COVID-19‡	165 (5.90)	177 (6.35)	173 (6.31)	123 (4.55)	116 (4.27)	754 (5.48)
**Presence of comorbidities (at least one)§**	2857 (100)	2825 (100)	2854 (100)	2866 (100)	2872 (100)	14 274 (100)
Yes	250 (8.75)	245 (8.67)	209 (7.32)	174 (6.07)	264 (9.19)	1142 (8.00)
No	2607 (91.25)	2580 (91.33)	2645 (92.68)	2692 (93.93)	2608 (90.81)	13 132 (92.00)

y – years

*The low-risk occupations include home-based workforce, students, homemakers, not working individuals.

†The medium-risk occupations include cultivators, agricultural workers, livestock, forestry, fishing, workers.

‡The high-risk occupations include frontline health care workers, sanitary workers, police workforce, workforce involved in transportation and essential industrial workers.

**§**Comorbidities include hypertension, diabetes, chronic renal failure, coronary heart disease, respiratory disease.

About 3211 (23%) of the participants had no formal schooling or had less than primary level schooling, 2862 (20%) had primary schooling, and 6951 (50%) attended middle or higher secondary standard. We categorized the participants’ occupations by the risk of exposure to COVID-19: high risk (frontline health care workers, sanitary workers, police, transportation, and essential industry workers); medium risk (agriculture and livestock workers, forestry, fishing) and low risk (home-based workforce, students, homemakers, not working individuals). Most of the study population belonged to medium or low risk of exposure to COVID-19 (**Table 1**).

### Reported COVID-19-like illness

Throughout all five rounds, about one-fourth of the study population reported experiencing COVID-19-like symptoms such as fever, chills, headache, myalgia, sore throat, runny nose, cough, or shortness of breath in the last four weeks. One in ten adults reported these COVID-19-like symptoms; however, about 4% of them had to miss work or required some medical attention for these COVID-19-like illnesses. Among the 135 hospitalizations reported due to COVID-19-like illness, about 91 (67.5%) cases were reported in the ≥50, 33 (24.5%) in the 18-49, and 11 (8%) in the 1-17-year-old age group (**Table 2**).

**Table 2:** COVID-19-like illness and its impact reported amongst the study participants

Morbidity characteristics	Round 1 (24 January 2021 to 20 February 2021), n (%)	Round 2 (21 February 2021 to 20 March 2021), n (%)	Round 3 (21 March 2021 to 24 April 2021), n (%)	Round 4 (25 April 2021 to 5 June 2021), n (%)	Round 5 (6 June 2021 to 26 June 2021), n (%)	All rounds, n (%)
**Participants per round**	n = 2857	n = 2825	N = 2854	n = 2866	n = 2872	n = 14 274
**Presence of COVID-19-like symptoms**						
1-17 y	125 (4.38)	185 (6.55)	124 (4.34)	143 (4.99)	147 (5.12)	724 (5.07)
18-49 y	237 (8.30)	275 (9.73)	239 (8.37)	288 (10.05)	334 (11.63)	1373 (9.62)
≥50 y	288 (10.08)	319 (11.29)	308 (10.79)	307 (10.71)	320 (11.14)	1542 (10.80)
**Missed work due to COVID-19-like symptoms**						
1-17 y	7 (0.25)	2 (0.07)	1 (0.04)	0 (0.00)	0 (0.00)	10 (0.07)
18-49 y	21 (0.74)	11 (0.39)	2 (0.07)	1 (0.03)	0 (0.00)	35 (0.25)
≥50 y	12 (0.42)	26 (0.92)	20 (0.70)	11 (0.38)	11 (0.38)	80 (0.56)
**Required medical attention for COVID-19-like Symptoms**						
1-17 y	8 (0.28)	1 (0.04)	1 (0.04)	1 (0.03)	0 (0.00)	11 (0.08)
18-49 y	22 (0.77)	13 (0.46)	2 (0.07)	0 (0.00)	0 (0.00)	37 (0.26)
≥50 y	13 (0.46)	28 (0.99)	21 (0.74)	10 (0.35)	10 (0.35)	82 (0.57)
**Required hospitalization for COVID-19 like symptoms**						
1-17 y	8 (0.28)	2 (0.07)	0 (0.00)	0 (0.00)	1 (0.03)	11 (0.08)
18-49 y	19 (0.67)	11 (0.39)	2 (0.07)	1 (0.03)	0 (0.00)	33 (0.23)
≥50 y	17 (0.60)	29 (1.03)	22 (0.77)	10 (0.35)	13 (0.45)	91 (0.64)

y – years

### Seroprevalence of SARS-CoV-2 antibodies

**Figure 2:**
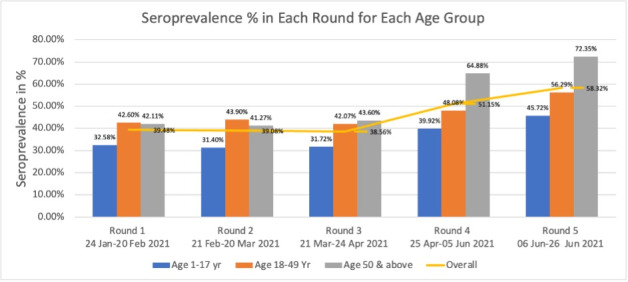
Seroprevalence (%) in all five rounds as per age groups.

Overall, 45% of the 14 274 participants tested positive for IgG antibodies, 51% of whom were males and 49% were females. In the first three rounds (January to April 2021), the overall seroprevalence remained constant at around 39%; however, it increased to 51% in round four (April to June 2021) and further to 58% in round five (June to June 2021). In round five, about 72% of the elderly individuals exhibited seropositivity. We observed the lowest seropositivity among the 1-17-year-old age group across all the five rounds (31%-46%, **Figure 2**).

### Factors associated with seropositivity for SARS-CoV-2

The distribution of seropositivity across sex, age, educational status, occupational category, history of COVID-19-like illness, contacts with suspected or confirmed COVID-19 cases, and history of COVID-19 vaccination were all statistically significant (*P* < 0.05). In univariate logistic regressions, the following factors were more strongly associated with seropositivity in descending order: being in contact with a suspected or confirmed case of COVID-19 (odds ratio (OR) = 7.15; 95% confidence interval (CI) = 4.2-12.14, *P* < 0.0001), receiving at least one dose of COVID-19 vaccine (OR = 3.13; 95% CI = 0.70-14.07, *P* < 0.0001), being ≥50 years old (OR = 1.97; 95% CI = 1.81-2.15, *P* < 0.0001), being in an occupation belonging to a high-risk category (OR = 1.92; 95% CI = 1.65-2.26, *P* < 0.0001), having a post-graduation educational status (OR = 1.59; 95% CI = 1.15-2.21, *P* < 0.0001). The odds of being seropositive was lower in males than females (OR = 0.93; 95% CI = 0.87-0.99, *P* = 0.032) (**Table 3**).

**Table 3:** Factors associated with seropositivity for SARS-CoV-2

Characteristic	Sero-negative	Sero-positive	*P*-value*	UR Analysis		
	**n (%)**	**n (%)**		**OR**	**95% CI**	***P*-value**
**Sex**	n = 7772	n = 6502				
Male	4094 (52.68)	3308 (50.88)		0.93	0.87-0.99	0.032
Female	3678 (47.32)	3194 (49.12)	0.032	Ref		
**Age group**	n = 7772	n = 6502				
1-17 y	2642 (33.99)	1489 (22.90)		Ref		
18-49 y	2981 (38.36)	2624 (40.36)		1.56	1.44-1.70	0.000
≥50 y	2149 (27.65)	2389 (36.74)	0.000	1.97	1.81-2.15	0.000
**Education category**	n = 7481	n = 6270				
No schooling or less than primary	1837 (24.56)	1374 (21.91)		Ref		
Primary	1590 (21.25)	1272 (20.29)		1.06	0.96-1.18	0.194
Secondary	3708 (49.57)	3243 (51.72)		1.17	1.07-1.27	0.000
Graduate	278 (3.72)	300 (4.78)		1.44	1.21-1.72	0.000
Postgraduate	68 (0.91)	81 (1.29)	0.000	1.59	1.15-2.21	0.000
**Occupation category**	n = 7481	n = 6270				
Low-risk category	2649 (35.41)	1593 (25.41)		Ref		
Medium-risk category	4483 (59.93)	4272 (68.13)		1.58	1.47-1.70	0.000
High-risk category	349 (4.67)	405 (6.46)	0.000	1.92	1.65-2.26	0.000
**Presence of COVID-19 like symptoms**	n = 7772	n = 6502				
Yes	1887 (24.28)	1752 (26.95)		1.15	1.07-1.24	0.000
No	5885 (75.72)	4750 (73.05)	0.000	Ref		
**Contact with suspected or confirmed COVID-19 case**	n = 7479	n = 6271				
Yes	26 (0.35)	140 (2.23)		7.15	4.2-12.14	0.000
No	7368 (98.52)	6067 (96.75)		Ref		
Don’t know	85 (1.14)	64 (1.02)	0.000	1.09	0.79-1.52	0.540
**COVID-19 vaccine at least dose 1**	n = 2637	n = 3141				
Yes	477 (18.09)	1116 (35.53)		3.13	0.70-14.07	0.000
No	2160 (81.91)	2025 (64.47)	0.000	Ref		

UR – univariate regression, OR – odds ratio, CI – confidence interval, y – years

*χ^2^ test.

## DISCUSSION

This is one of the first studies from Western India reporting a gradual increase in population prevalence of SARS-CoV-2 infection in a rural, geographically defined area in the Pune district using a repeated serosurvey study design between January and June 2021.

Crucially, our study provided information on the prevalence of infection over a period when the incidence of COVID-19 infections was rising in Pune. While serosurveys are not reliable for providing information on disease burden, repeated serosurveys are an important epidemiological tool for estimating the burden based on the force of infection and can be used by public health managers to design disease prevention and mitigation strategies.

The overall seroprevalence was 46% across all five rounds, remaining at around 39% for the first three rounds and then increasing to 58% by the fifth round. Our results differ from those of a repeated seroprevalence study in North India conducted between August and October 2020, which reported a decline in seroprevalence (28.39% to 24.08%) [20]. Our findings correspond to a relatively steady level of reported COVID-19 cases from January 2021 to March 2021 followed by an increase from April 2021 to June 2021 [2]. Although COVID-19 vaccination was introduced in February 2021 for frontline workers and in April 2021 for the elderly population, the number of vaccinated individuals was not high enough to significantly affect our seroprevalence estimates. While associations between infection and disease cannot be directly estimated from our study, it can be used to develop modelling scenarios to estimate true burden of SARS-CoV-2 and predict the pandemic’s future course. We found that children <18 years of age had significantly lower seroprevalence than the older age group. These results might help policymakers with deciding on modalities and opportunities for keeping schools and educational institutes open.

The overall seroprevalence (45%) of SARS-CoV-2 antibodies in the rural Pune district was slightly lower than that found in an earlier study conducted in the urban areas of the Pune district, which found a seroprevalence of 51.5% in August 2020 [21]. Another study from the rural district of south India also found a lower seroprevalence in the rural area when compared to the urban areas of South India [22]. However, the increase in seroprevalence to 58% in the fifth round of our study shows that the rate of spread of SARS-CoV-2 is most likely similar in rural and urban areas and that preventive measures are needed equally for both populations.

The increased seropositivity in individuals >50 years of age compares well with the Delhi study using repeated serosurveys [20]. We are yet to fully understand why the elderly are at a higher risk of contracting the infection (not the disease), though certain mechanisms such as a progressive and relatively linear increase in nasal cavity volume with increasing age coupled with an age-dependent decrease of nasal resistance have been proposed as possible causes [23].

A female preponderance in our study is consistent with the aforementioned Delhi study [20], but not with a nationwide Indian study that did not find any age or sex differentials in antibody positivity [24]. Thus, this association needs to be validated further with more data.

While we did find that working in an environment with a higher risk of exposure to the virus was significantly associated with the presence of IgG antibodies, the absolute numbers are too low to arrive at any definitive conclusion. This factor may be important during early stages of a pandemic, but when most of the population is already infected, as seen in our study in the fifth round, occupational risk is likely to have a limited role in infection transmission. As expected, contacts of suspected or confirmed COVID-19 cases had a higher risk of testing seropositive for SARS-CoV-2 IgG antibodies. This only reiterates the importance of contact tracing as a crucial tool to reduce the burden of COVID-19.

This cross-sectional study has several limitations. First, the study team visited all the contiguous households in selected villages. Since the second wave (with the Delta B.1.617.2 variant) peaked and villagers were apprehensive, we may have missed sampling some individuals who were eligible for study participation. However, the relatively large number of individuals included in our study can compensate for this sampling bias to some extent. Second, we did not perform COVID-19 confirmatory real-time reverse transcription polymerase chain reaction (RT-PCR) tests in symptomatic individuals, so we may have missed the current infection rate. We cannot distinguish between seropositivity due to natural infections and COVID-19 vaccines in vaccinated individuals.

This study is one of the earliest conducted population-based serosurveillance studies (with a serial-surveillance approach) in rural India. Our findings provide evidence regarding the spread of the infection and its implications in the rural area. Only 5.7% of approached participants refused participation, despite fear of acquiring COVID-19. We credit this success to the massive stakeholder engagement activity performed in the field thus indicating the positive role of such engagements in community-based studies like this.

While the presence of IgG antibodies indicates infection, antibody-induced protection to disease can be determined by the presence of neutralising antibodies, which we did not assess. This can be investigated, along with correlation of cell-mediated immunity in a follow-up study in this population. Additionally, the duration of seroprevalence and its association with re-infection and disease severity needs to be investigated, considering the emergence of newer variants of SARS-CoV-2. An extensive stakeholder engagement drive was undertaken to drive successful recruitment and implementation in this study. The team built trust with local communities through these meetings, with continued engagement during fieldwork and data collection helping build rapport and create a sense of responsibility for the study. Community members provided feedback on the serological test results to individual study participants and informed the communities of overall seroprevalence rates.

## CONCLUSIONS

Our findings suggest that seroprevalence of SARS-CoV-2 was high in last two rounds (April to June 2021), which coincide with the second wave (with the Delta B.1.617.2 variant) in the country. One in three children and one in two adults showed antibodies for SARS-CoV-2. The suspected or confirmed case of COVID-19 emerged as the significant factor strongly associated with the seropositivity followed by the COVID-19 vaccination. The appropriate and timely community engagement was key for establishing sentinel serosurveillance during the pandemic. Initiating early serosurveillance and continuously following the population is recommended for informed public health response.
